# Supplementary material for: Ultimate thin vertical p–n junction composed of two-dimensional layered molybdenum disulfide
Source: Nat Commun. 2015 Mar 24;6:6564. doi: 10.1038/ncomms7564 (PMC4383009; doi:10.1038/ncomms7564)
Supplement: Supplementary Information — Supplementary Figures 1-4, Supplementary Table 1, Supplementary Notes 1-4 and Supplementary References [file ncomms7564-s1.pdf]

Supplementary Figures

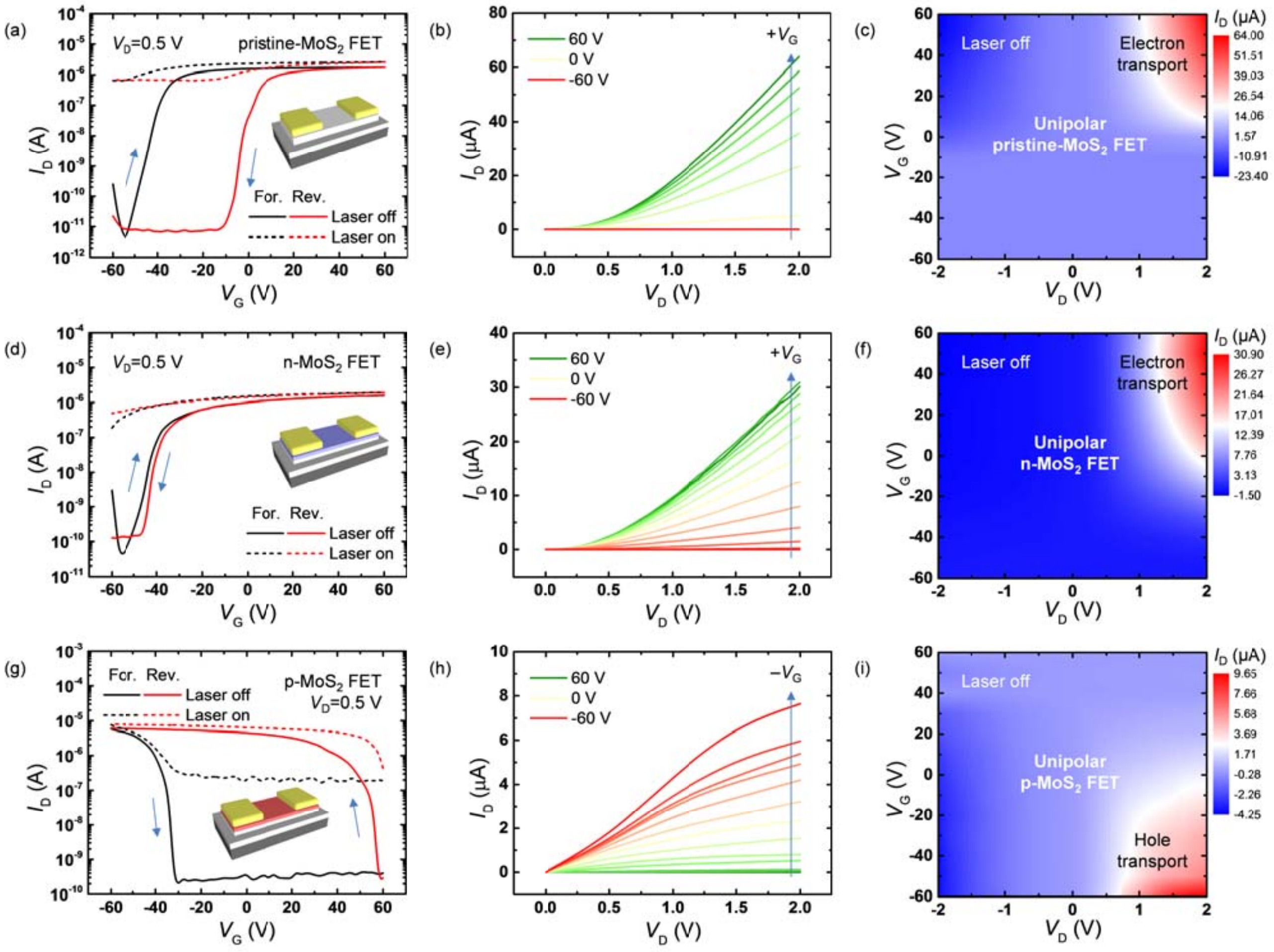

Supplementary Figure 1 **Effects of chemical doping on the performances of various MoS_2 FET devices.** (a, d, g) Transfer characteristics of the pristine-MoS_2, n-MoS_2, and p-MoS_2 FETs and their photoresponse during both forward and reverse sweeps. The corresponding insets show schematic diagrams of the pristine-MoS_2, n-MoS_2, and p-MoS_2 FETs. (b, e, h) Output characteristics of the pristine-MoS_2, n-MoS_2, and p-MoS_2 FETs for varying V_G levels, from 60 to –60 V, with steps of 10 V. (c, f, i) Channel current mappings of the pristine-MoS_2, n-MoS_2, and p-MoS_2 FETs at various V_D (from –2 to 2 V) and V_G (from –60 to 60 V) levels indicate the presence of unipolar carrier transport under dark conditions.

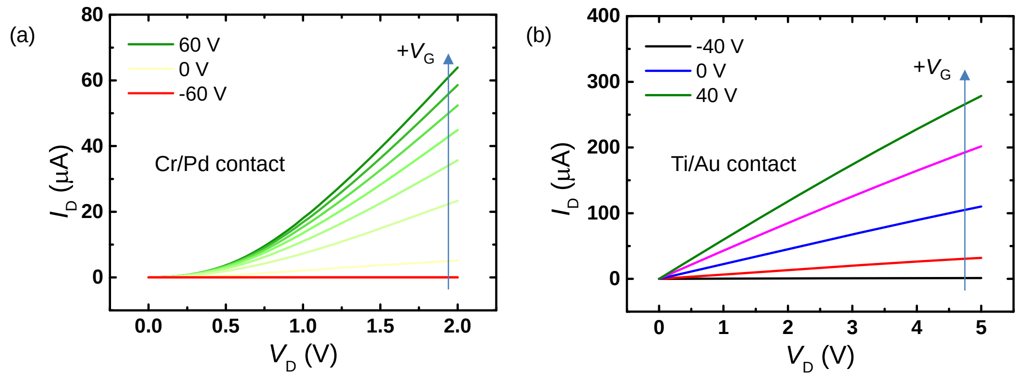

Supplementary Figure 2 **Comparison of output characteristics of back-gate MoS_2 FETs.** The MoS_2 FETs were fabricated with few-layer, pristine MoS_2 flakes using (a) Cr/Pd (5 nm / 50 nm) electrode contacts, and (b) Ti/Au (3 nm / 50 nm) electrode contacts. The data shown in (a) is from Fig. S1(b) in this work, and V_G varies from 60 to –60 V, along steps of 10 V. The data shown in (b) is from Fig.6(a) in our previous work[4], and replotted in the same form of (a) for comparison. V_G varies from –40 to 40 V, along steps of 20 V.

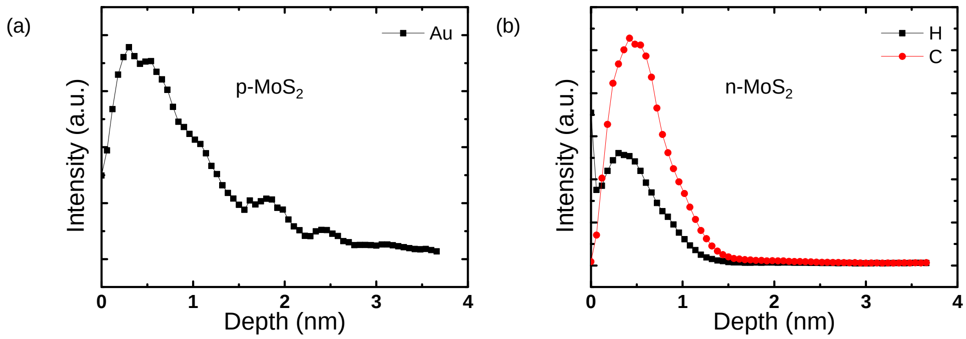

Supplementary Figure 3 **Doping profile in MoS_2 flakes.** Chemical elements distribution including (a) Au in p-MoS_2 flake, and (b) H and C in n-MoS_2 flake were measured as a function of depth by SIMS.

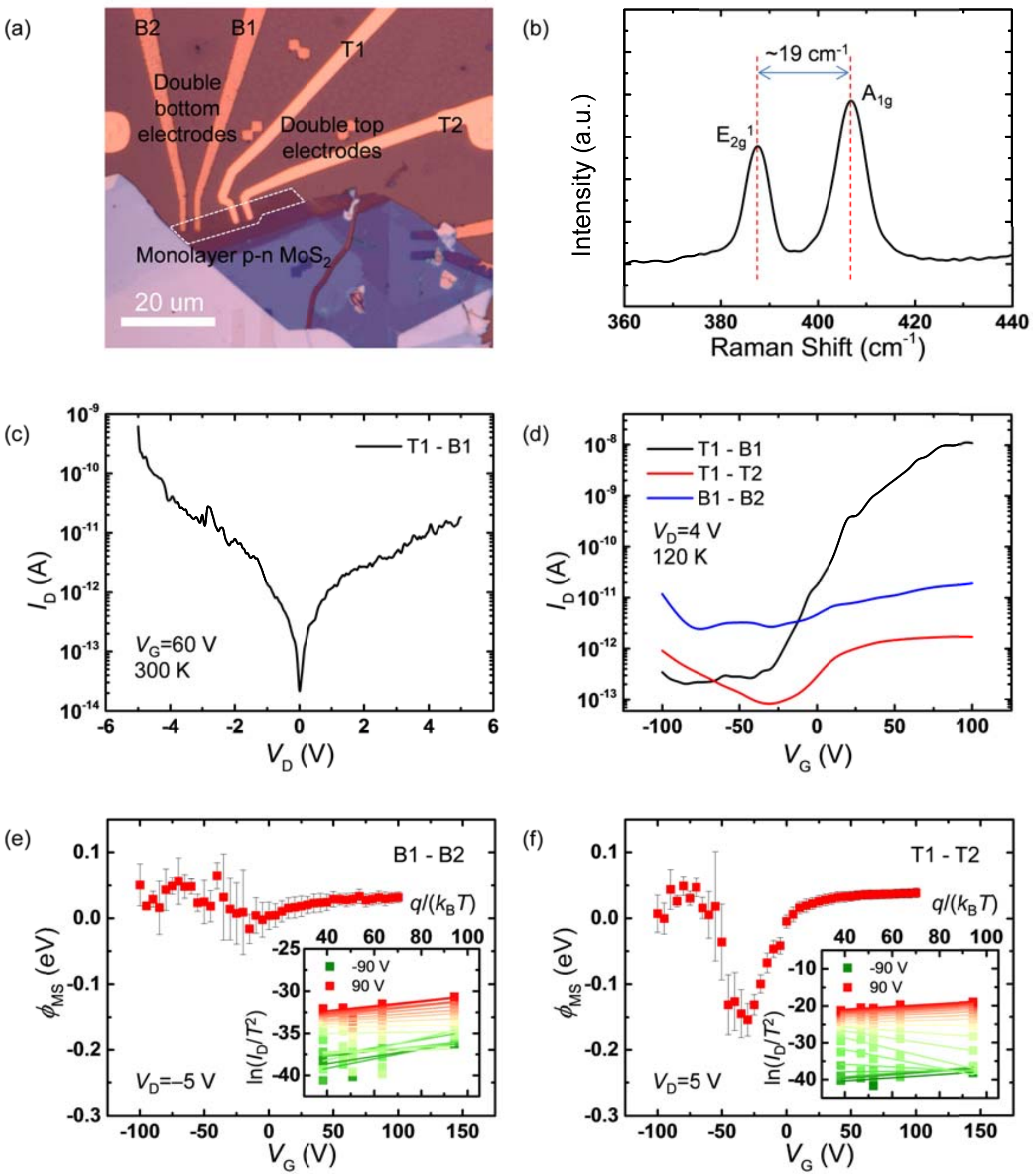
(a)
B2
B1
T1
T2
Double bottom electrodes
Double top electrodes
Monolayer p-n MoS2
20 um
(b)
~19 cm-1
A1g
E2g1
Intensity (a.u.)
Raman Shift (cm-1)
(c)
T1 - B1
VG=60 V
300 K
ID (A)
VD (V)
(d)
T1 - B1
T1 - T2
B1 - B2
VD=4 V
120 K
ID (A)
VG (V)
(e)
B1 - B2
q/(kBT)
-90 V
90 V
ln(ID/T2)
VD=-5 V
ϕMS (eV)
VG (V)
(f)
T1 - T2
q/(kBT)
-90 V
90 V
ln(ID/T2)
VD=5 V
ϕMS (eV)
VG (V)

Supplementary Figure 4 **Carrier transport in vertical monolayer MoS_2 p-n junction.** (a) Optical microscopy image illustrates a monolayer MoS_2 p-n junction with double top electrodes (T1 and T2) and double bottom electrodes (B1 and B2). (b) Raman spectrum illustrates E_{2g}^1 and A_{1g} peaks, suggesting a monolayer structure of MoS_2. (c) Output characteristics of monolayer MoS_2 p-n junction measured by employing T1 and B1 electrodes at room temperature. (d) Transfer characteristics of monolayer MoS_2 p-n junction measured by employing T1 and B1 electrodes at 120 K, as well as the individual transfer characteristics obtained from the top surface (by employing T1 and T2 electrodes) and from the bottom surface (by employing B1 and B2 electrodes). (e, f) Metal–semiconductor barrier height as a function of gate voltage obtained from the bottom surface (by employing B1 and B2 electrodes) and the top surface (by employing T1 and T2 electrodes). The corresponding insets are the temperature-dependent current characteristics and their corresponding linear fits for various V_G levels. The error bars indicate standard deviation of the linear fits in the insets.

Supplementary Tables

Supplementary Table 1. Comparison of solar energy conversion performance of MoS_2 and its hybrid systems.

Device type	Active layer thickness	PCE	FF	EQE
This work: Vertical MoS_2 p-n junction	11 nm	0.4%	0.22	/
Lateral MoS_2 p-n junction[5]	60 nm	/	/	~7000% (biased)
MoS_2-Au[6]	220 nm (MoS_2 layer)	1.8%	0.55	52%
MoS_2-graphene[7]	0.9 nm	1.0%	0.6 (assumed)	/
MoS_2-WS_2[7]	1.2 nm	1.5%	0.6 (assumed)	/
MoS_2-WSe_2[8]	1.2 nm	0.2%	/	1.5%
MoS_2-Si[9]	0.6 nm (MoS_2 layer)	5.2%	0.57	~60%

(Here PCE is the power conversion efficiency, FF is the fill factor, EQE is the external quantum efficiency. The lateral MoS_2 p-n junction[8] was not tested as the solar cells in a solar simulator.)

Supplementary Note 1

Comparative study of chemically doped n-MoS_2 and p-MoS_2 FETs

The effects of the chemical doping on carrier transport and device performance were investigated by fabricating and comparing the performances of p-MoS_2, n-MoS_2, and pristine-MoS_2 FETs. The few-layer MoS_2 flakes were obtained by mechanical exfoliation from bulk crystals and carefully selected to have an approximate thickness of 10 nm. After transfer to a p-type Si substrate coated with a 90 nm thick thermal oxide layer, the MoS_2 flakes were chemically doped to form p-type semiconductors by introducing $AuCl_3$ or to form n-type semiconductors by introducing BV. Pristine samples were reserved as reference samples. Symmetric metal electrodes composed of Cr/Pd (5 nm / 50 nm) were deposited to create the MoS_2 FETs. The drain-to-source current (I_D) was characterized as a function of the drain and gate voltages (V_D and V_G) using a semiconductor parameter analyzer. A monochromator (655 nm, 15 mW) was combined with electrical measurements to test the photoresponse.

The output characteristics (I_D-V_D) were measured over a wide range of V_D (from –2 to 2 V) and V_G (from –60 to 60 V), and the transfer characteristics (I_D-V_G) were examined in both dark and laser-illuminated environments, as shown in Fig. 1. The pristine-MoS_2 FET showed n-type carrier transport with an on/off ratio of ~10^5. In the current-on state (V_G = 60 V), the metal–semiconductor contact (Pd/Cr/pristine-MoS_2) displayed a symmetric and non-linear I-V relation at small V_D, suggesting Schottky-like contact (see Fig. 1(a)–(c)). Similarly, the n-MoS_2 FET (or n^+-MoS_2 FET, to distinguish this device from the pristine-MoS_2 FET) also showed electron transport properties with an on/off ratio of ~10^4. The metal–semiconductor contact (Pd/Cr/n-MoS_2) displayed

asymmetric Schottky-like behavior in the current-on state (V_G = 60 V) and showed a reverse saturation current (I_{sat}) under a negative V_D (see Fig. 1(d)–(f)). By contrast, the carrier transport properties of the p-MoS_2 FET were exactly opposite these properties. The device showed hole transport properties with an on/off ratio of ~10^5. The metal–semiconductor contact (Pd/Cr/p-MoS_2) displayed asymmetric Schottky-like behavior in the current-on state (V_G = –60 V), and I_{sat} was observed under a positive V_D (see Fig. 1(g)–(i)). Our results clearly demonstrated that both $AuCl_3$ and BV could effectively modulate the Fermi level and alter the carrier transport type in the MoS_2 films.

It has been demonstrated that the electron injection and hole injection in ambipolar WSe_2 FETs can be improved by reducing the barrier height with proper metal electrodes[1], and this can also apply to unipolar MoS_2 FETs[2,3]. In our previous work, the MoS_2 FETs with few-layer pristine MoS_2 flake showed very good Ohmic contact to Ti/Au (3 nm / 50 nm) electrodes in the same back-gate structure[4]. Therefore, it was reasonable to infer that the Schottky-like contact behavior observed from the pristine-MoS_2 FET in this work was attributed from the larger work function of Cr (4.5 eV) compared to Ti (4.3 eV), as shown in Fig. 2.

Compared to the pristine device (40 V), the hysteresis window (ΔV_{hys}) between forward and reverse sweep decreased upon introducing n-type doping (5 V), but increased upon introducing p-type doping (90 V). The origin of hysteresis was charge-trapping and de-trapping phenomenon, which was under a kinetic equilibrium condition to maintain charge neutrality. The ΔV_{hys} of a uniform pristine MoS_2 film can be expressed as $\Delta V_{hys} = qAn_{trap}/C_{ox}$, where q is the electronic charge, A is the planar area, n_{trap} is the trap density, and C_{ox} is the oxide capacitance. Since the pristine MoS_2 has electron

transport propriety, n_{trap} represents the electron traps in this work. For a few-layer MoS_2 film chemically doped from the top surface, the doping-dependent ΔV_{hys} can be further deduced as

$$\Delta V_{hys} = \frac{qA\int_0^d n_{trap}(z)dz}{C_{ox}}, \quad (1)$$

where d is the MoS_2 film thickness. Since MoS_2 differed from graphene in that the current flow path in the few-layer MoS_2 was near the top surface in a back-gate structure[3], the electron traps near the top surface were considered as the major traps for generating hysteresis. Therefore, the n-type doping would induce more electrons near the top surface and fill those electron traps, giving rise to a reduced n_{trap} and thereby a small ΔV_{hys}. By contrast, the p-type doping would de-trap the electrons which were captured by those electron traps at the equilibrium of pristine MoS_2, giving rise to an increased n_{trap} and thereby a large ΔV_{hys}.

Supplementary Note 2

Comparison of optoelectronic performance

We did a literature survey on solar energy harvesting devices based on MoS_2 p-n junction and MoS_2 hybrid systems with other materials, including lateral MoS_2 p-n junction[5], MoS_2-Au[6], MoS_2-graphene[7], MoS_2-WS_2[7], MoS_2-WSe_2[8], and MoS_2-Si[9] systems. A comparison of optoelectronic performance was shown in Table 1. To the best of our knowledge, the MoS_2-Si[9] and MoS_2-Au[6] systems provided the best solar energy conversion efficiencies so far. However, the contribution from bulk Si and bulk Au layers in those two systems should also be noted for the comparison. Our previous work, the

lateral MoS_2 p-n junction[5], was not tested as the solar cells in a solar simulator. Its optoelectronic performance, such as the ultrahigh external quantum efficiency (EQE), was measured at a certain biasing condition (V_D=1.5 V, V_G=−40 V) under a laser illumination (350 nm of wavelength). However, it was still worth including this device in the comparison due to the geometrically comparative structure. Compared to the other types of solar energy harvesting systems, the vertical MoS_2 p-n homogeneous junction demonstrated in this work provided a reasonable and comparable performance. We believe the performance can be further improved by optimizing device parameters, including the layer thickness, electrode layout, doping agent and concentration etc.

Supplementary Note 3

Doping profile in MoS_2 flakes

We experimentally measured the thickness limit for a vertical MoS_2 p-n junction to be 3 nm (4 layers). The chemical doping depth along the direction perpendicular to the layers was estimated to be 1.5 nm (2 layers) for both p- and n-type doping. In order to confirm the doping depth, a direct observation of doping profile in MoS_2 flakes by using secondary ion mass spectroscopy (SIMS) was made, as shown in Fig. 3. The doping depth was found to be 2 nm for p-type doping (Au atoms in $AuCl_3$), and to be 1.5 nm for n-type doping (C and H atoms in BV).

Supplementary Note 4

Carrier transport in vertical monolayer MoS_2 p-n junction

Another vertical monolayer MoS_2 p-n junction was fabricated with the same process as described in the Methods of manuscript. It had double top electrodes (T1 and T2) and double bottom electrodes (B1 and B2) in order to confirm the carrier transport type on the top and bottom surfaces, respectively, as shown in Fig. 4(a). The bottom electrodes were made of Cr/Pd/Cr (5 nm / 50 nm / 5 nm), and the top electrodes were made of Cr/Pd (5 nm / 50 nm), in order to provide symmetric metal contact to MoS_2 flake.

The layer number of monolayer MoS_2 was confirmed by Raman spectroscopy, as shown in Fig. 4(b). The difference of Raman shift between E_{2g}^1 and A_{1g} peaks was measured as ~19 cm^{-1}, suggesting a monolayer structure of MoS_2 flake[10].

Output characteristics of vertical monolayer MoS_2 p-n junction was measured by employing T1 and B1 electrodes under vacuum conditions (10 mTorr) at room temperature. It showed "reversed" current rectification in which a tunneling-dominated large current was observed at the reversed bias, as shown in Fig. 4(c). This was consistent with the electrical behavior of monolayer MoS_2 p-n junction as shown in the manuscript (see Fig. 5(e) in the main manuscript), suggesting the good reproducibility and reliability of the vertical MoS_2 p-n junction in this work.

In order to reduce the external thermal interference, transfer characteristics of vertical monolayer MoS_2 p-n junction were measured at the low-temperature (120 K) by employing T1 and B1 electrodes, as shown in Fig. 4(d). The monolayer MoS_2 p-n junction showed unipolar electron transport over a wide V_G range. This was consistent with the electrical behavior of another device shown in the manuscript (see Fig. 6(d) in the main manuscript), and indicated the overwhelming of n-type doping through the entire MoS_2 flake.

The carrier transport type on the top and bottom surfaces can be confirmed individually. The transfer characteristics on the top surface of MoS_2 (referred as p-MoS_2) were measured by employing T1 and T2 electrodes. Similarly, the transfer characteristics on the bottom surface of MoS_2 (referred as n-MoS_2) were measured by employing B1 and B2 electrodes. As we demonstrated in the manuscript, the p- and n-type doping can effectively modulate the Fermi level and alter the carrier transport type of MoS_2 (see Fig. 1(d) and (g)). However, the individual transfer characteristics on both the top and bottom surfaces showed electron-dominated carrier transport, suggesting the compromise of p-type doping and the overwhelming of n-type doping (see Fig. 4(d)). In other words, the p-n junction cannot be properly formed by the chemically doping in monolayer MoS_2. Here we also note that even the overall monolayer MoS_2 showed n-type carrier transport, the dominance of electron transport in each individual transfer curves was not as clear as that shown in the few-layer MoS_2 (see Fig. 1(d)). The degradation of n-type doping in the monolayer MoS_2 suggested that the n-type doping was also compromised partially by the p-type doping, and therefore its doping effect was suppressed. This also agreed with our theory.

To quantitatively analyze the metal–semiconductor contact condition, the metal–semiconductor barrier height (ϕ_{MS}) was obtained by applying a temperature-dependent test. For the carrier transport through a metal–semiconductor barrier, the current-voltage relation can be written as[4]

$$I_D = AA^*T^2 \exp\left(\frac{-\phi_{MS}}{k_B T}\right)\left[\exp\left(\frac{-qV_D}{k_B T}\right) - 1\right], \qquad (2)$$

where A is the area of the contact junction, A^* is the effective Richardson constant, q is the electronic charge, k_B is Boltzmann constant, and T is the temperature. Under a high

V_D, the contact at the drain end was reversely biased [$\exp(-qV_D/k_BT)<<1$], and I_D became proportional to $T^2\exp(-\phi_{MS}/k_BT)$. A linear relation between $\ln(I_D/T^2)$ and q/k_BT can be plotted for various V_G levels, and the gate-dependent ϕ_{MS} for a given V_D was estimated from the slope of each curve, as shown in Fig. 4(e) and (f). Both ϕ_{MS} obtained from the top surface (Pd/Cr/p-MoS_2) and from the bottom surface (Pd/Cr/n-MoS_2) showed a strong dependence on V_G, reflecting a gate-controlled metal–semiconductor barrier modulation[4]. The maximum value of ϕ_{MS} obtained from both the top and bottom metal-semiconductor interfaces were about 40 meV at the positive V_G, which was in agreement with the electrical behavior of a Schottky-like junction as we discussed previously (see Fig. 2).

Supplementary References

1. Das, S. & Appenzeller, J. WSe_2 field effect transistors with enhanced ambipolar characteristics. *Appl. Phys. Lett.* **103**, 103501 (2013).
2. Das, S., Chen, H.-Y., Penumatcha, A. V. & Appenzeller, J. High performance multilayer MoS_2 transistors with scandium contacts. *Nano Lett.* **13**, 100–105 (2013).
3. Das, S. & Appenzeller, J. Where does the current flow in two-dimensional layered systems? *Nano Lett.* **13**, 3396–3402 (2013).
4. Li, H.-M. *et al.* Metal-semiconductor barrier modulation for high photoresponse in transition metal dichalcogenide field effect transistors. *Sci. Rep.* **4**, 4041 (2014).
5. Choi, M. S. *et al.* Lateral MoS_2 p-n junction formed by chemical doping for use in high-performance optoelectronics. *ACS Nano* **8**, 9332–9340 (2014).
6. Shanmugam, M., Durcan, C. A. & Yu, B. Layered semiconductor molybdenum disulfide nanomembrane based Schottky-barrier solar cells. *Nanoscale* **4**, 7399–7405 (2012).
7. Bernardi, M., Palummo, M. & Grossman, J. C. Extraordinary sunlight absorption and one nanometer thick photovoltaics using two-dimensional monolayer materials. *Nano Lett.* **13**, 3664–3670 (2013).
8. Furchi, M. M., Pospischil, A., Libisch, F., Burgdorfer, J. & Muller, T. Photovoltaic effect in an electrically tunable van der Waals heterojunction. *Nano Lett.* **14**, 4785–4791 (2014).
9. Tsai, M.-L. *et al.* Monolayer MoS_2 heterojunction solar cells. *ACS Nano* **8**, 8317–8322 (2014).
10. Lee, C. *et al.* Anomalous lattice vibrations of single- and few-layer MoS_2. *ACS Nano* **4**, 2695–2700 (2010).
